# Influence of cerebral blood flow on volumetric loss related to Alzheimer’s disease

**DOI:** 10.1590/1980-5764-DN-2023-0004

**Published:** 2023-09-25

**Authors:** Maria Izaura Sedoguti Scudeler Agnollitto, Renata Ferranti Leoni, Maria Paula Foss, Julia Palaretti, Marcela Cayres, Vitor Pansarim, Julio Cesar Nather, Maria Clara Zanon Zotin, Eduardo Ferrioli, Nereida Kilza Lima, Antonio Carlos dos Santos, Julio Cesar Moriguti

**Affiliations:** 1 Universidade de São Paulo, Faculdade de Medicina de Ribeirão Preto, Ribeirão Preto SP, Brazil.; 2 Universidade de São Paulo, Faculdade de Filosofia Ciências e Letras de Ribeirão Preto, Ribeirão Preto SP, Brazil.

**Keywords:** Alzheimer Disease, Cognitive Dysfunction, Magnetic Resonance Imaging, Cerebrovascular Circulation, Doença de Alzheimer, Disfunção Cognitiva, Imagem por Ressonância Magnética, Circulação Cerebrovascular

## Abstract

CBF measured with Arterial Spin Labeling (ASL) obtained by Magnetic Resonance Imaging (MRI) may become an important biomarker by showing changes in early stages of AD, such as in the prodromal stage of Mild Cognitive Impairment (MCI).

**Objective:**

Verify the correlation between atrophy and CBF in patients with MCI and mild phase ADD, to demonstrate whether changes in CBF can be considered as vascular biomarkers in the diagnosis of the DA continuum.

**Methods:**

11 healthy volunteers, 16 MCI and 15 mild ADD were evaluated. Images of the brain were acquired, including CBF measured with Arterial Spin Labeling (ASL).

**Results:**

When comparing MCI with control, a reduction in normalized CBF was observed in left posterior cingulate (estimated difference -0.38; p=0.02), right posterior cingulate (estimated difference -0.45; p=0.02) and right precuneus (estimated difference -0.28; p <0.01); also increase in normalized CBF in right upper temporal pole (estimated difference 0.22; p=0.03). It was also observed that in MCI, the smaller the gray matter volume, the smaller the CBF in the left posterior cingulate; as well as the greater the cerebrospinal fluid volume, consequent to the encephalic volumetric reduction associated with atrophy, the greater the CBF in the right superior temporal pole. When comparing controls, MCI and mild AD, in relation to the other variables, no other correlations were observed between CBF and atrophy.

**Conclusion:**

In patients with MCI, the reduction of CBF in the left posterior cingulate correlated with gray matter atrophy, as well as the increase of CBF in the right upper temporal pole correlated with an increase in cerebrospinal fluid consequent to the encephalic volumetric reduction associated with atrophy, demonstrating the influence of CBF in AD related brain atrophy. These findings position CBF as a possible vascular biomarker for early-stage AD diagnoses.

## RESUMO

A imagem por ressonância magnética (IRM) pode se tornar um importante biomarcador ao mostrar alterações nos estágios iniciais da doença de Alzheimer (DA).

**Objetivo:**

Sendo a atrofia cerebral um importante biomarcador de neurodegeneração na DA, o presente estudo foi realizado com o objetivo de verificar se há correlação entre atrofia e fluxo sanguíneo cerebral (FSC) em pacientes com diagnóstico de CCL e demência da doença de Alzheimer (DDA) leve, com o objetivo de revelar se as alterações no FSC podem ser consideradas possíveis biomarcadores vasculares no diagnóstico do *continuum* da DA.

**Métodos:**

Foram avaliados 11 voluntários saudáveis, 16 CCL e 15 DDA leve. Imagens do cérebro foram adquiridas em um equipamento de 3 T, incluindo imagens ponderadas em T1 de alta resolução para avaliação anatômica e *Arterial Spin Labeling* (ASL) para a quantificação de FSC.

**Resultados:**

Quando comparado CCL com controle, observou-se redução no FSC normalizado em cingulado posterior esquerdo (diferença estimada de -0,38; p=0,02), cingulado posterior direito (diferença estimada de -0,45; p=0,02) e precúneo direito (diferença estimada de -0,28; p <0,01); e aumento de FSC normalizado no polo temporal superior direito (diferença estimada de 0,22; p=0,03). No CCL, quanto menor o volume da substância cinzenta, menor o FSC no cingulado posterior esquerdo; quanto maior o volume de fluido cerebroespinhal, consequente à redução volumétrica encefálica, maior o FSC no polo temporal superior direito.

**Conclusão:**

Nos pacientes com diagnóstico de CCL, a redução de FSC no cingulado posterior esquerdo apresentou correlação com atrofia da substância cinzenta, assim como o aumento de FSC no polo temporal superior direito apresentou correlação com o aumento de fluido cerebroespinhal, demonstrando a provável influência do FSC na atrofia encefálica relacionada à DA.

## INTRODUCTION

The increase in life expectancy resulting from socioeconomic development and medical advances has led to rapid population aging worldwide. The impact of this process on health is noticed in the increase of chronic-degenerative diseases. Dementia is one of the most serious as it causes a progressive loss of autonomy and independence, becoming a problem for individuals, their families, and public health^1^. It is estimated that currently, around 50 million people live with dementia worldwide and this number could triple by 2050^2^.

Dementia is a disorder characterized by progressive cognitive decline severe enough to reduce an individual’s ability to perform usual occupational or social activities. It can compromise cognitive domains such as memory, language, attention, executive function, learning, visuospatial ability, and abstract thinking^3^. The most common cause of dementia, accounting for 60 to 80% of cases, is Alzheimer’s disease (AD), a primary, progressive, and irreversible neurodegenerative disorder that causes cognitive, particularly memory, and functional decline. Its prevalence doubles every five years in individuals aged 65 to 85 years^4^.

The most prevalent form of Alzheimer’s disease dementia (ADD) is the sporadic, late-onset form, which occurs in individuals over 65 years of age, caused by a complex association of genetic and environmental factors^5^. The mechanisms by which risk factors influence AD are not well defined, but it is believed that they lead to the development and establishment of pathophysiological changes^1^. The main pathophysiological markers of AD are extracellular amyloid plaques and intracellular neurofibrillary tangles^2,6^ that spread in the brain following a consistent pattern evidenced by atrophy mainly in the temporal lobes, entorhinal cortex, hippocampus, and posterior cingulate gyrus^6^.

The pathophysiological changes begin decades before the AD clinical manifestations. The progression of clinically imperceptible brain alterations to established cognitive deficit is called the “Alzheimer’s disease *continuum”* and describes three phases: pre-clinical, mild cognitive impairment, and dementia^7^. In the pre-clinical phase, pathophysiological changes are already present but still do not cause clinical manifestations due to the action of compensatory mechanisms^8^. Mild cognitive impairment (MCI) due to AD is characterized by the presence of cognitive alterations, most commonly in recent memory, but that does not interfere with the individual’s ability to perform daily activities and may be considered a prodromal stage^9,10^. In ADD, cognitive changes become more evident, impairing the individual’s previous functionality^3^. The most common initial cognitive manifestation is the impairment of recent memory, with difficulty acquiring and remembering new information, which insidiously worsens over months to years, compromising other cognitive domains with the evolution of the disease^11^.

Magnetic resonance imaging (MRI) of the brain can be considered a potential biomarker by showing alterations in the pre-clinical and prodromal phases before established dementia. Early diagnosis at these stages allows for better clarification of the pathophysiology and the study of new disease-modifying therapies, in addition to allowing counseling, treatment planning, and care for the patient and their family members/caregivers^12^.

Brain MRI data to assess AD can be qualitative and quantitative. The present work evaluated such data in healthy individuals and patients diagnosed with MCI and mild ADD. Since brain atrophy is an important biomarker of neurodegeneration in AD, the present study was conducted to verify whether there is a correlation between atrophy and cerebral blood flow (CBF) in patients diagnosed with MCI and mild ADD. We aimed to assess whether changes in CBF can be considered as possible vascular biomarkers in identifying the AD *continuum*. The hypothesis was that, in comparison with the control group, atrophy and CBF alteration would be observed in the MCI group, such alterations would be presented more prominently in the mild ADD group, and there could be a correlation between atrophy and CBF changes.

## METHODS

A cross-sectional, descriptive, and comparative study was carried out, in which qualitative and quantitative characteristics of brain MRI of healthy individuals, and patients diagnosed with MCI and mild ADD were evaluated. The study was approved by the Clinics Hospital of the Faculty of Medicine of Ribeirão Preto (HC-FMRP) Ethics Committee. Participants were clarified about the nature of the study and the assessments to which they would be submitted. All of them signed the Informed Consent Form before participation. Patients with an established dementia diagnosis had their terms signed by their carers.

A total of 42 elderly over 60 years of age were included in the study, being patients diagnosed with MCI and mild ADD assisted in the Geriatric healthcare service at HC-FMRP, in addition to healthy individuals from the community. Their mean age was 76.89, standard deviation (±) of 6.84 years, and most of them were female (54.76%).

The following exclusion criteria were adopted: dementia whose etiology was not AD; moderate and severe ADD; a sensory deficit that could interfere with the neuropsychological assessment; previous history of cerebrovascular disease of any etiology; neurological diseases such as Parkinson’s disease, hydrocephalus, infections, epilepsy, expansive lesions, brain trauma; psychiatric illnesses such as schizophrenia, major depressive disorder, bipolar affective disorder, personality disorder; and characteristics that would contraindicate the performance of MRI of the brain.

Individuals were submitted to neuropsychological assessment, applied by a neuropsychologist, with a battery of sensitive tests to assess the healthy elderly and the elderly with cognitive impairment. The assessment was developed specifically for the present study by an experienced neuropsychologist, covering multiple cognitive domains. All tests were validated for application in the studied population.

The evaluation began with the 15-item Geriatric Depression Scale (GDS-15) application for tracking depressive symptoms^13,14^. It was adopted as a cutoff score (of 5/6) to exclude individuals with probable depressive symptoms, which could interfere with the final result of the neuropsychological assessment. At this stage, Mini-Mental State Examination (MMSE) data were also obtained for global cognitive assessment^15,16^, and the Hachinski Ischemic Scale to verify the possibility of associated cerebrovascular disease^17^.

Afterward, the following tests were applied: Three Words and Three Shapes Test (3W3S), which evaluates learning and episodic memory^18,19^; Rey’s Auditory-Verbal Learning Test (RAVLT), which assesses learning, evocation, and episodic memory^20,21^; Mattis Dementia Rating Scale (MDRS) that indicates general cognitive status including attention, initiation/perseveration, construction, conceptualization and memory^22,23^; Five-Digit Test (FDT) that measures the speed of cognitive processing, the ability to focus, sustain and reorient attention, inhibitory control, mental flexibility and the ability to deal with interference^24^; Boston Naming Test (BNT) that focuses on naming and semantic memory^25^; and the Weschler Intelligence Rating Scale Vocabulary (WIRSV), which assesses language, lexical knowledge, ease of speech elaboration and semantic memory, in addition to estimating intelligence coefficient^26^. Finally, Pfeffer’s Functional Activities Questionnaire (PFAQ) was applied to examine functionality^27,28^. Individuals with scores above 5 were classified as having a functional disability.

Two experienced neuropsychologists discussed the test battery results. Individuals without cognitive and functional deficits were classified as healthy. If no change in functionality was found but a documented cognitive deficit, the patient was diagnosed with MCI. If established functional incapacity associated with cognitive deficit was found, the patient was diagnosed with ADD. We considered the diagnosis of possible AD for the study. Subjects underwent the Clinical Dementia Ratio (CDR) classification^29^ and the Clinical Dementia Ratio-Sum of Boxes (CDR-SOB)^30^ to assess cognitive impairment severity. Patients with moderate or severe dementia were excluded from the sample. The results of the neuropsychological evaluation classified the individuals into three groups: control, MCI, and mild ADD.

Images from all participants were acquired in a 3 Tesla Magnetic Resonance equipment, Philips Achieva 3T X-series (Philips Medical Systems, Best, Netherlands), in a 32-channel phased-array coil, from the radiology service of HC-FMRP. Sequences were performed to obtain images according to a protocol developed specifically for the present study, which included:

- T1-weighted images, 3D gradient-echo volumetric, magnetization prepared rapid gradient echo, FLAIR 3D (fluid-attenuated inversion recovery), with high contrast, acquired in the sagittal plane and reconstructed in the three orthogonal planes, isotropic voxel; with TR/TE=7.4/3.5 ms; voxel size=1x1x1 mm^3^; flip angle=8°; FOV=240x240 mm^2^; 160 to 180 slices.
- Volumetric T2-weighted images with 3D fluid suppression, FLAIR, acquired in the sagittal plane and reconstructed in the three orthogonal planes, isotropic voxel; with TR/TE=5000/327 ms; 1600 ms inversion time; voxel size=1x1x1 mm^3^; FOV=240x240 mm^2^; 160 to 180 slices.
- 2D pseudo-continuous arterial spin labeling (PCASL) axial images acquired with echo-planar imaging (EPI) readout with the following parameters: excitation angle=90°, matrix=80x80, FOV=240x240 mm², 20 slices, slice thickness= 5 mm, TR/TE=4000/14 ms, labeling time (LT)=1650 ms, post-labeling delay (PLD)=1525 ms, 50 control/label pairs and duration of 6 minutes and 40 seconds.

Images were evaluated by two experienced neuroradiologists, who did not have access to the individuals’ clinical data, at two different moments with an interval of one month. It was performed in a different random order for each examiner in each analysis to obtain the following qualitative data: medial temporal atrophy (MTA)^31,32^, entorhinal cortex atrophy (ERICA)^33^ and posterior cortical atrophy (PCA)^34^.

Matlab/SPM12 and Hippodeep softwares were used to process the images and obtain the following quantitative data: left and right hippocampal volume, hippocampal asymmetry index, white and gray matter volumes, and cerebral spinal fluid volume.

PCASL images were processed by the InBrain Medical Physics team using the softwares Matlab/SPM12 and ASL Toolbox. First, the images were pre-processed to reduce variations not associated with the baseline in the subject’s resting state and prepare the data for processing. Afterward, PCASL images were motion-corrected, and those with movements exceeding 2 mm or 2° were excluded. Next, they were co-registered with anatomical images, which were segmented to obtain gray matter (GM), white matter (WM), and cerebrospinal fluid (CSF) masks. PCASL images were then spatially smoothed with a Gaussian filter (4-mm full-width at half-height) to improve the signal-to-noise ratio.

After pre-processing, control and label images were subtracted to obtain perfusion-weighted images. CBFe quantification was performed following the model^35^:

$$CBF=\frac{6000\cdot \lambda \cdot \left(SI_{control}-SI_{label}\right)\cdot \frac{PLD}{e^{T1\;blood}}}{2\cdot \alpha \cdot T_{1,blood}\cdot SI_{PD}\cdot \;\;(1-e^{-\frac{\tau }{T1\;blood}}}\left[\frac{mL\cdot min}{100g}\right]$$

Where λ is the brain/blood partition coefficient in mL/g; *SI*_control_ and *SI*_label_ are the time averages of the signal strength in the control and label images, respectively; T1_blood_ is the blood longitudinal relaxation time in seconds; *a* is the labeling efficiency; *SI*_PD_ is the signal strength of a proton density-weighted image, and *t* is the labeling time. All values used in this study are those recommended in the literature^35^.

CBF maps were then normalized to the standard Montreal Neurological Institute (MNI) coordinate system space (spatial resolution=2x2x2 mm³, 79x95x79 matrix), and the CBF values were calculated for different brain regions.

The first analysis performed was the agreement among neuroradiologists regarding the qualitative variables, using the Weighted Kappa Coefficient. Qualitative data were described using absolute frequencies and percentages. Quantitative data were described using mean, standard deviation, minimum, median, and maximum values. An Ordinal Multimodal Logistic Regression Model was used for comparisons between groups regarding qualitative data. Analysis of covariance (ANCOVA) and Tukey’s post-test were performed for comparisons between groups regarding quantitative data. All models were adjusted for age, gender, education, marital status, systemic arterial hypertension, diabetes, smoking, atrial fibrillation, and use of anticholinesterase drugs. For all comparisons, a significance level of 5% was adopted.

Data were organized and analyzed using the softwares SAS (version 9.2, SAS, Cary, North Carolina, USA) and R (version 4.0.0, The R-Project for Statistical Computing, Open License Software).

## RESULTS

Forty-two individuals were included in the study: 11 controls, 16 MCI, and 15 mild ADD. The main characteristics of all individuals are shown in Table 1. GDS, MMSE, Hachinski and Pfeffer data are described in the Supplementary Material.

**Table 1.** Characteristics of individuals.

Characteristics	Control (%)	MCI (%)	Mild ADD (%)	Total (%)
Individuals number	11	16	15	42
Age				
Mean age ± SD (years)	73.45 (6.65)	78.31 (7.19)	78.93 (6.68)	76.89 (6.84)
Median (years)	74	79.5	80	77
Minimum age (years)	66	67	67	66
Maximum age (years)	88	93	91	91
Sex				
Female	4 (36.36)	11 (68.75)	8 (53.33)	23 (54.76)
Male	7 (63.64)	5 (31.25)	7 (46.67)	19 (45.24)
Educational level				
Up to 8 years	4 (36.36)	10 (62.5)	15 (100)	29 (69.95)
More than 8 years	7 (63.64)	6 (37.5)	0 (0)	13 (30.95)
Marital status				
With partner	10 (90.91)	6 (37.5)	5 (33.33)	21 (50)
Without partner	1 (9.09)	10 (62.5)	10 (66.67)	21 (50)
Comorbidities				
SAH	6 (54.55)	10 (62.5)	10 (66.67)	26 (61.9)
DM^|^	4 (36.36)	6 (37.5)	5 (33.33)	15 (35.71)
AF	2 (18.18)	1 (6.25)	1 (6.67)	4 (9.52)
Smoking	1 (9.09)	0 (0)	4 (26.67)	4 (11.9)
Anticholinesterase	1 (9.09)	2 (12.5)	11 (33.33)	14 (33.33)

Abbreviations: MCI: mild cognitive impairment; ADD: Alzheimer’s disease dementia; SD: standard deviation; SAH: systemic arterial hypertension; DM: diabetes mellitus; AF: Atrial fibrillation.

Comparative analyzes of qualitative data between control, MCI, and mild ADD groups are described in Table 2. Comparing MCI with control, there was a difference in the left MTA (odds ratio [OR] 9.63; p-value [p]=0.02), right MTA (OR 6.14; p=0.04), and left ERICA (OR 9.09; p=0.04). No difference was observed regarding the right ERICA and PCA. There was no difference in the qualitative assessment when comparing MCI with mild ADD. However, a difference was found in the left MTA (OR 0.07; p=0.03) between control and mild ADD.

**Table 2.** Comparison of qualitative data.

Qualitative data	OR	p-value	95%CI
Left MTA			
MCI-control	9.63	0.02	(1.48–62.50)
MCI-mild ADD	0.71	0.74	(0.10–5.21)
Control-mild ADD	0.07	0.03	(0.01–0.82)
Right MTA			
MCI-control	6.14	0.04	(1.01–37.41)
MCI-mild ADD	1.12	0.91	(0.15–8.41)
Control-mild ADD	0.18	0.15	(0.02–1.90)
Left ERICA			
MCI-control	9.09	0.04	(1.08–76.49)
MCI-mild ADD	0.90	0.93	(0.09–8.61)
Control-mild ADD	0.10	0.10	(0.01–1.57)
Right ERICA			
MCI-control	7.91	0.07	(0.81–76.94)
MCI-mild ADD	1.04	0.98	(0.11–9.84)
Control-mild ADD	0.13	0.15	(0.01–2.14)
PCA			
MCI-control	2.03	0.55	(0.20–20.49)
MCI-mild ADD	0.72	0.78	(0.08–6.92)
Control-mild ADD	0.36	0.48	(0.02–6.19)

Abbreviations: OR: odds ratio; MTA: medial temporal atrophy; MCI: mild cognitive impairment; ADD: Alzheimer’s disease dementia; ERICA: entorhinal cortex atrophy; PCA: posterior cortex atrophy.

Quantitative data from brain volumes and asymmetry index are described in Table 3. Comparative analyzes of quantitative data between control, MCI, and mild ADD groups are described in Table 4. Controls presented a white matter volume of 82,442 mm^3^ greater than mild ADD (p=0.04; 95%CI 1,035.99–163,848.00). No difference was observed between groups concerning left and right hippocampal volume, asymmetry index, gray matter volume, and cerebrospinal fluid volume.

**Table 3.** Quantitative data of brain volumes and asymmetry index.

Quantitative data^*^	Control	MCI	Mild ADD
Left hippocampal (mm^3^)	2,948.78 (513.71)	2,594.28 (534.91)	1932.24 (548.56)
Right hippocampal (mm^3^)	3,168.49 (577.03)	2,671.29 (525.88)	2115.96 (565.97)
Asymmetry index	7.09 (4.91)	2.94 (9.13)	9.42 (26.46)
White matter (mm^3^)	612,589.75 (81,232.34)	532,738.58 (76,818.72)	488,755.23 (64,158.98)
Gray matter (mm^3^)	424,009.39 (55,805.51)	386,247.16 (68,360.05)	366,231.77 (69,705.98)
Cerebrospinal fluid (mm^3^)	381,165.46 (135,721.61)	459,352.49 (108,432.87)	489,200.23 (119,382.05)

Abbreviations: MCI: mild cognitive impairment; ADD: Alzheimer’s disease dementia. Notes:

*Quantitative data presented in average (standard deviation).

**Table 4.** Comparison of quantitative data.

Quantitative data	Estimated difference	p-value	95%CI
Left hippocampal (mm^3^)			
MCI-control	-311.38	0.15	(-710.81–88.06)
MCI-mild ADD^+^	-8.51	1.00	(-471.01–454.00)
Control-mild ADD	302.87	0.35	(-228.14–833.88)
Right hippocampal (mm^3^)			
MCI-control	-428.59	0.06	(-872.96–15.78)
MCI-mild ADD	-69.78	0.94	(-584.31–444.76)
Control-mild ADD	358.81	0.31	(-231.93–949.55)
Asymmetry index			
MCI-control	-1.24	0.99	(-19.98–17.50)
MCI-mild ADD	-2.20	0.97	(-23.89–19.50)
Control-mild ADD	-0.96	0.99	(-25.87–23.95)
White matter (mm^3^)			
MCI-control	-46,855.00	0.16	(-108,090.00–14381.00)
MCI-mild ADD	35,587.00	0.44	(-35,318.00–106,492.00)
Control-mild ADD	82,442.00	0.04	(1,035.99–163,848.00)
Gray matter (mm^3^)			
MCI-control	-3,192.84	0.99	(-57,133.00–50,747.00)
MCI-mild ADD	-26,703.00	0.55	(-89,160.00–35,754.00)
Control-mild ADD	-23,510.00	0.70	(-95,218.00–48,197.00)
Cerebrospinal fluid (mm^3^)			
MCI-control	72,742.00	0.32	(-49,404.00–194,888.00)
MCI-mild ADD	-14,246.00	0.97	(-155,679.00–127,187.00)
Control-mild ADD	-86,988.00	0.40	(-249,367.00–75,392.00)

Abbreviations: MC: mild cognitive impairment; ADD: Alzheimer’s disease dementia.

**Figure 1.**
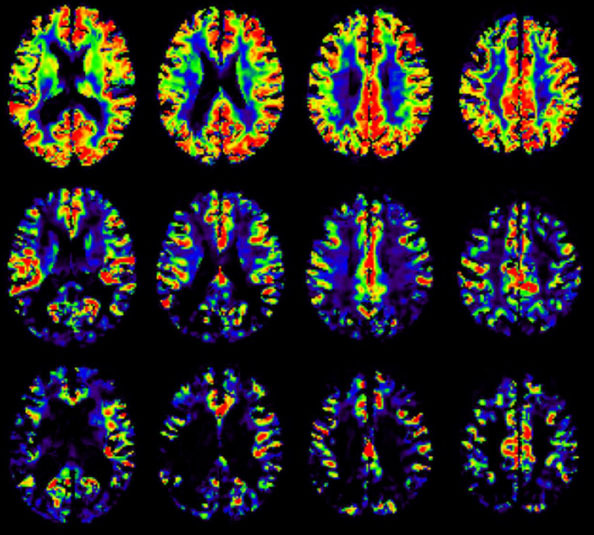
Quantitative cerebral blood flow maps. Four slices of three representative participants of the groups: Control (upper row), Mild cognitive impairment (middle row), and Alzheimer’s disease dementia (bottom row). Values are from 0 (blue) to 100 mL.100g/min (red).

CBF data from brain regions were obtained, and these data were normalized to the value of the whole brain. Figure 1 shows representative CBF maps of three participants. Table 5 describes CBF absolute values, and Table 6, the normalized values of regions with significant differences among groups. Table 7 shows the comparative analysis of normalized CBF. Compared to controls, CBF reduction was noted in MCI in the left posterior cingulate (estimated difference of -0.38; p=0.02), right posterior cingulate (estimated difference of -0.45; p=0.02), and right precuneus (estimated difference of -0.28; p<0.01).

**Table 5.** Cerebral blood flow absolute values.

Brain region	Control	MCI	Mild ADD
Left posterior cingulate (mL.min/100g)	51.99 (17.07)	34.21 (16.09)	40.33 (18.60)
Right posterior cingulate (mL.min/100g)	51.54 (20.61)	32.79 (15.98)	42.16 (20.41)
Right precuneus (mL.min/100g)	45.48 (14.56)	31.76 (11.46)	37.76 (16.80)

Abbreviations: MC: mild cognitive impairment; ADD: Alzheimer’s disease dementia. Notes: Values in average (standard deviation).

**Table 6.** Cerebral blood flow normalized values.

Brain region	Control	MCI	Mild ADD
Left posterior cingulate	1.28 (0.23)	0.92 (0.29)	1.03 (0.35)
Right posterior cingulate	1.24 (0.30)	0.89 (0.32)	1.07 (0.37)
Right precuneus	1.12 (0.18)	0.87 (0.17)	0.95 (0.25)

Abbreviations: MC: Mild cognitive impairment; ADD: Alzheimer’s disease dementia. Notes: Values in average (standard deviation).

**Table 7.** Comparison of normalized cerebral blood flow quantitative data.

Brain region	OR	p-value	95%CI
Left posterior cingulate			
MCI-control	-0.38	0.02	(-0.70; -0.05)
MCI-mild ADD	-0.29	0.15	(-0.66; 0.08)
Control-mild ADD	-0.09	0.87	(-0.34; 0.52)
Right posterior cingulate			
MCI-control	-0.45	0.02	(-0.82; -0.08)
MCI-mild ADD	-0.31	0.20	(-0.74; 0.12)
Control-mild ADD	0.14	0.76	(-0.35; 0.64)
Right precuneus			
MCI-control	-0.28	<0.01	(-0.49; -0.07)
MCI-mild ADD	-0.05	0.88	(-0.29; 0.19)
Control-mild ADD	0.23	0.11	(-0.041; 0.51)

Abbreviations: OR: odds ratio; MC: mild cognitive impairment; ADD: Alzheimer’s disease dementia.

**Figure 2.**
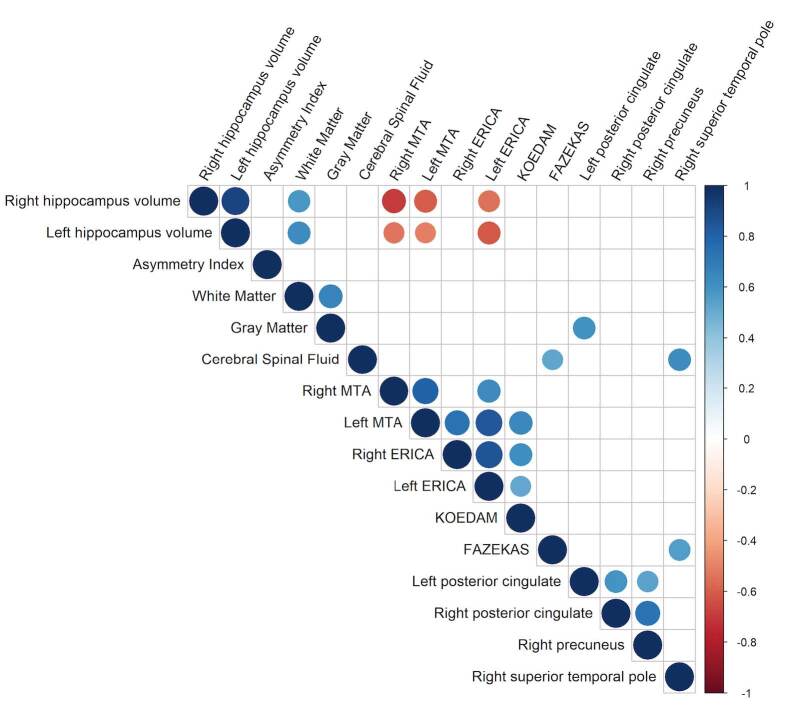
Mild cognitive impairment Spearman’s correlations.

Abbreviations: MTA: medial temporal atrophy; ERICA: entorhinal cortex atrophy.

Spearman’s correlation was performed between the variables that described atrophy and CBF. When observing the MCI group, gray matter volume correlated positively with CBF in the left posterior cingulate. Cerebrospinal fluid was positively correlated with CBF in the right superior temporal pole. CBF in the right posterior cingulate and left and right precuneus correlated positively with each other. The correlations are described in Figure 2.

**Figure 3.**
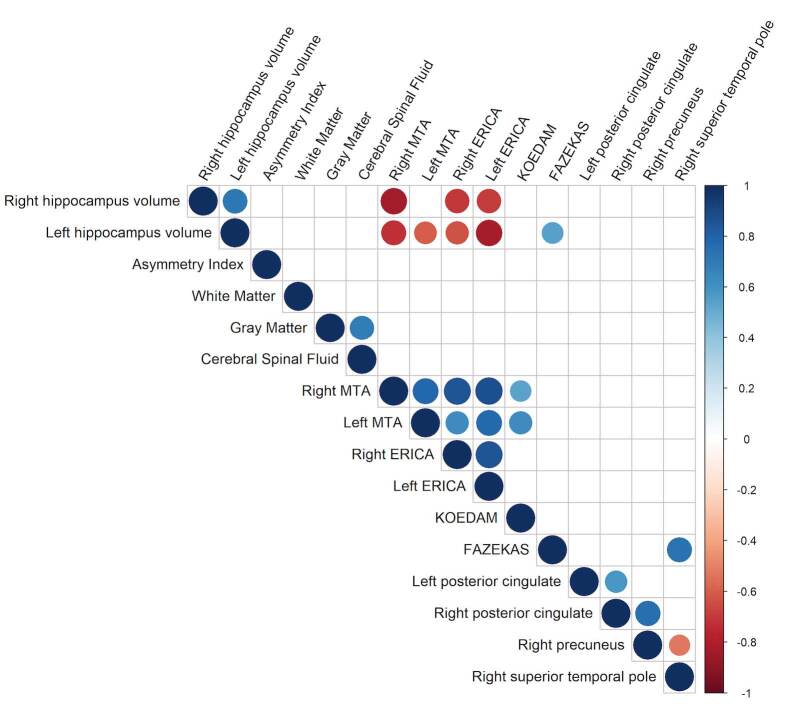
Mild Alzheimer’s disease dementia Spearman’s correlations.

Abbreviations: MTA: medial temporal atrophy; ERICA: entorhinal cortex atrophy.

When observing the mild ADD group, atrophy measurements did not correlate with CBF measurements in the evaluated regions. CBF at the right superior temporal pole correlated negatively with CBF at the right precuneus. Correlations are described in Figure 3.

## DISCUSSION

Regarding qualitative data, when comparing MCI with control, the former was more likely to have higher scores in left MTA, right MTA, and left ERICA. The hippocampus and entorhinal cortex were the structures that presented early changes associated with the neurodegeneration process^36,37^. They could be seen, in advance, in patients diagnosed with MCI, being documented by higher scores in MTA and ERICA, and consistent with the findings of the present study. When comparing mild ADD with control, the former was more likely to have higher scores in left MTA. It may also be associated with the hippocampus being a structure that presents early changes associated with degeneration^36,37^. We found no significant differences in other structures when comparing these groups, maybe because of the small sample size. 

Regarding quantitative data, it was observed that the control had a greater volume of white matter than mild ADD. A previous study described the same alteration, with a reduction predominantly in the medial temporal, inferior parietal, and middle frontal regions in patients diagnosed with ADD. This finding seems to be related to cortical atrophy with demyelination of fibers^38^. Another study showed that white matter alteration could be detected in early stages of AD, in MCI, and even in pre-clinical stages, especially in the temporal and parietal region, suggesting that such alteration is an early characteristic of the disease^39^.

Although the volume of the left and right hippocampus and gray matter progressively decreased from the control group to the MCI and mild ADD group, there was no significant difference when comparing the groups. In addition, there was an increase in cerebrospinal fluid volume observed progressively from the control group to the MCI and mild ADD group, but with no significant difference in the comparison. Such findings may be related to brain atrophy and consequent increase in ventricular volume^40^; no differences were observed, possibly because of the small sample size. A previous study showed the same findings, except for presenting a significant difference in the reduction of the left hippocampus volume when compared control group with mild ADD^41^, which can be explained by the asymmetric regional cerebral atrophy characteristic of AD as described in other studies^42,43^.

A study that compared controls with MCI and mild ADD showed that the gray matter volume was reduced in the MCI group, particularly in the medial and lateral temporal lobes^44^. Such a finding was not observed in the present work, most likely owing to the small sample.

Some studies demonstrated hypoperfusion in several brain areas, but the most consistent findings were reported in the posterior cingulate and precuneus^45^, corroborating the present work. Regarding the quantitative data of normalized CBF, when comparing MCI with the control group, the first showed a reduction in CBF in regions of the left posterior cingulate, right posterior girdle, and right precuneus. A study published in 2019 demonstrated that such alteration performed well in differentiating MCI from control and mild ADD from control, but not in differentiating MCI from mild ADD. It could be explained by hypoperfusion being more accentuated in the early stages of the disease, before established dementia, when such a change is not as evident^46^.

Another study showed that MCI and mild ADD had similar perfusion alterations, in particular, hypoperfusion in the posterior cingulate and precuneus, in addition to hyperperfusion in the medium temporal^47^, and this latter finding was not evidenced in the present work.

Overall, the most consistent finding was hypoperfusion in the posterior cingulate from the pre-clinical phase to the MCI and dementia onset, can be considered a functional biomarker of the disease^48^. Longitudinal studies observed posterior cingulate hypoperfusion in healthy elderly who developed subsequent cognitive impairment, which may be useful for predicting conversion^49,50^. A study published in 2017 analyzed patients diagnosed with mild ADD followed for two years and found that the rate of CBF decline, particularly in the posterior regions, may had a value associated with cognitive decline; that is, changes in CBF could also be considered prognostic markers in AD^36^. Areas with CBF alteration demonstrated atrophy associated with cognitive manifestation in other studies^47-49^.

Some regions presented increased CBF in patients with MCI and mild ADD, but no significant difference between the groups. Other studies showed hyperperfusion in some regions in these phases and could be justified as a compensatory mechanism for neurodegeneration^51,52^. It revealed no significant differences compared to the present work, probably as a result of the small sample size,

Although all individuals were carefully selected, the absence of an amyloid biomarker and a definitive pathological diagnosis does not exclude other underlying abnormalities or mixed pathophysiological processes. The cross-sectional design of the study does not allow the observation of conversion of patients to MCI and mild ADD, which would only be possible with longitudinal follow-up of the individuals in the sample. Another limitation of the cross-sectional design is that it does not allow for establishing a cause-and-effect relationship between the findings.

The relatively small sample size makes comparing and identifying differences between groups complex. The large number of variables and comparisons included in the study limits the findings’ strength. Additional studies are essential to highlight the crucial role of combining different brain MRI techniques to investigate different neurodegeneration aspects and increase the AD continuum’s diagnostic accuracy.

The study demonstrated that in patients with MCI, the reduction of CBF in the left posterior cingulate correlated with gray matter atrophy, as well as the increase of CBF in the right upper temporal pole correlated with an increase in cerebrospinal fluid, evidencing the likely influence of CBF on AD-related brain atrophy. These findings position CBF as a possible vascular biomarker for early-stage AD diagnoses.
